# Supplementary material for: Magnetic Particle Imaging-Guided Thermal Simulations for Magnetic Particle Hyperthermia
Source: Nanomaterials (Basel). 2024 Jun 20;14(12):1059. doi: 10.3390/nano14121059 (PMC11206764; doi:10.3390/nano14121059)
Supplement: Supplementary file 1 [file nanomaterials-14-01059-s001.zip › nanomaterials-2998661-supplementary.pdf]

nanomaterials

Supplementary Materials

Magnetic Particle Imaging-Guided Thermal Simulations for Magnetic Particle Hyperthermia

Hayden Carlton[a], Nageshwar Arepally[b], Sean Healy[a], Anirudh Sharma[a], Sarah Ptashnik[c], Maureen Schickel[c], Matt Newgren[d], Patrick Goodwill[d], Anilchandra Attaluri[b], Robert Ivkov[a,e-g*]

[a] Department of Radiation Oncology and Molecular Radiation Sciences, the Johns Hopkins University School of Medicine, Baltimore, MD, USA
[b] Department of Mechanical Engineering, School of Science, Engineering, and Technology, The Pennsylvania State University – Harrisburg; Middletown, PA, USA
[c] Materialise NV, Leuven, Belgium
[d] Magnetic Insight Inc., Alameda, CA, USA
[e] Department of Oncology, Sydney Kimmel Comprehensive Cancer Center, Johns Hopkins University School of Medicine, Baltimore, MD, USA
[f] Department of Mechanical Engineering, Whiting School of Engineering, Johns Hopkins University, Baltimore, MD, USA
[g] Department of Materials Science and Engineering, Whiting School of Engineering, Johns Hopkins University, Baltimore, MD, USA
* Correspondence: rivkov1@jhmi.edu

MPI and CT Co-Registered Images of Mice

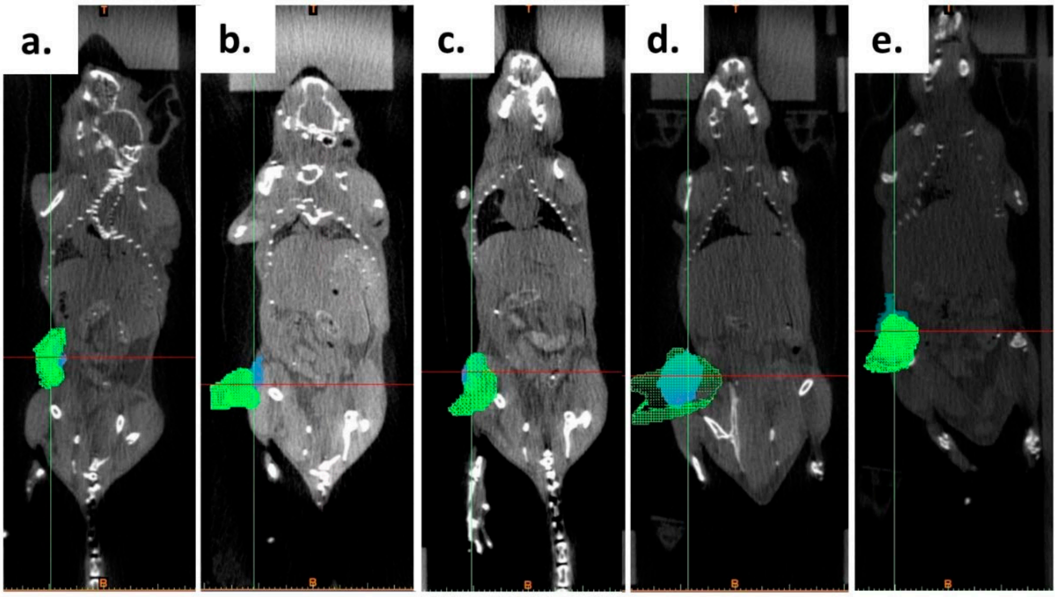

Figure S1. Co-registered MPI/CT images of a. Tumor 1; b. Tumor 2; c. Tumor 3; d. Tumor 4; e. Tumor 5. The teal regions are the segmented subcutaneous tumors, while the green regions are the segmented MPI scans. .

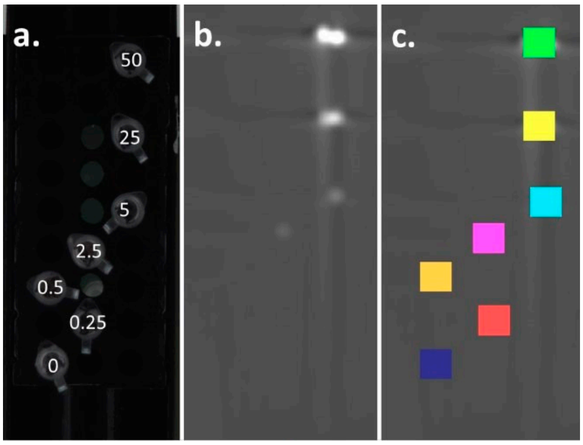

Figure S2. a. Calibration samples in well plate sample holder; b. MP image of the calibration sample; c. Mimics segmentation of each calibration sample.

Table S1. MNP content of calibration samples with estimated thermal output and MPI GV. .

Mass (µg)	Conc. (mgFe/mL)	Q_{MNP} (W/m^3)	Max GV Avg	Max GV Std. Dev.
0	0	0	1024.00	0.00
5	0.25	124000	1025.33	0.58
10	0.5	248000	1029.00	1.00
50	2.5	1240000	1044.33	0.58
100	5	2480000	1063.33	2.52
500	25	12400000	1200.67	16.07
1000	50	24800000	1288.33	5.69

Experimental Rectal Temperature Data

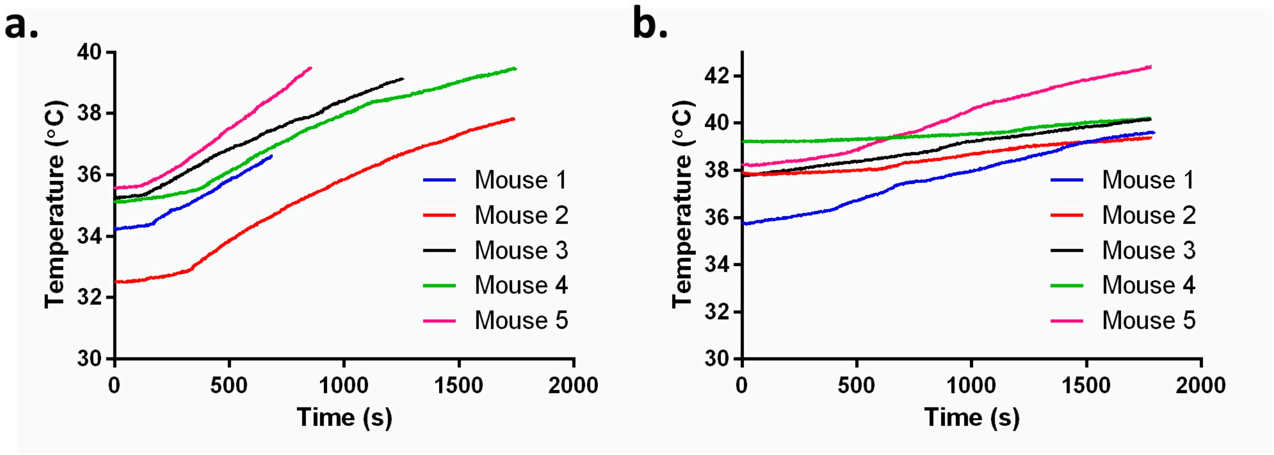

Figure S3. Rectal temperature data from each mouse for the a. Continuous; and b. Pulsed heating trials.

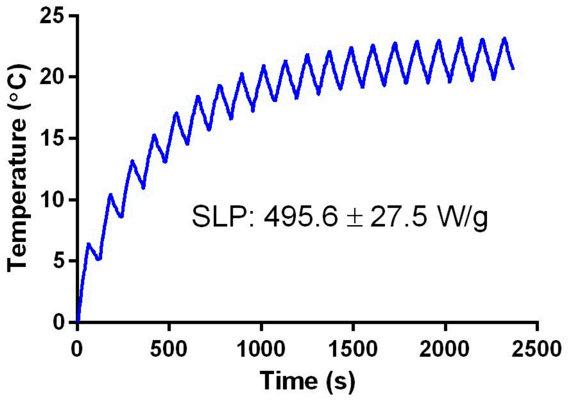

Figure S4. Temperature vs time plot used to calculate SLP using the Pulsed Box Lucas methodology.

Processing Imaging Data for COMSOL

Software Needed

1. Mimics Innovation Suite with FEA Module
2. COMSOL Multiphysics with Bioheat and AC/DC Modules

Datasets Needed

1. MPI scan of MNP calibration samples at selected settings.
2. MPI scan of tumored mouse with injected MNPs, at the same settings used for calibration samples, with at least three MNP fiducials placed within the scan volume along the sample holder
3. CT scan of tumored mouse with the same orientation and fiducial placement as the MPI scan

Co-Registration

1. Import anatomical and MPI scans into Mimics, where they can both be seen under the Project Management tab.
2. Select and view the anatomical image.
3. Under the Image tab, select Landmark Registration.
4. A window will appear with a 3x2 grid of images, where the anatomical image planes will be visible in the left column. Under Dataset 2, import the MPI scan.
5. Find your first fiducial in both the anatomical and MPI scans, select the "Add", and select the location of the fiducial on both sets of images scans.
6. Repeat for the other fiducials. (Figure S5).
7. Select OK, and Mimics will generate a text file containing the transformation matrix and save it in your selected directory.

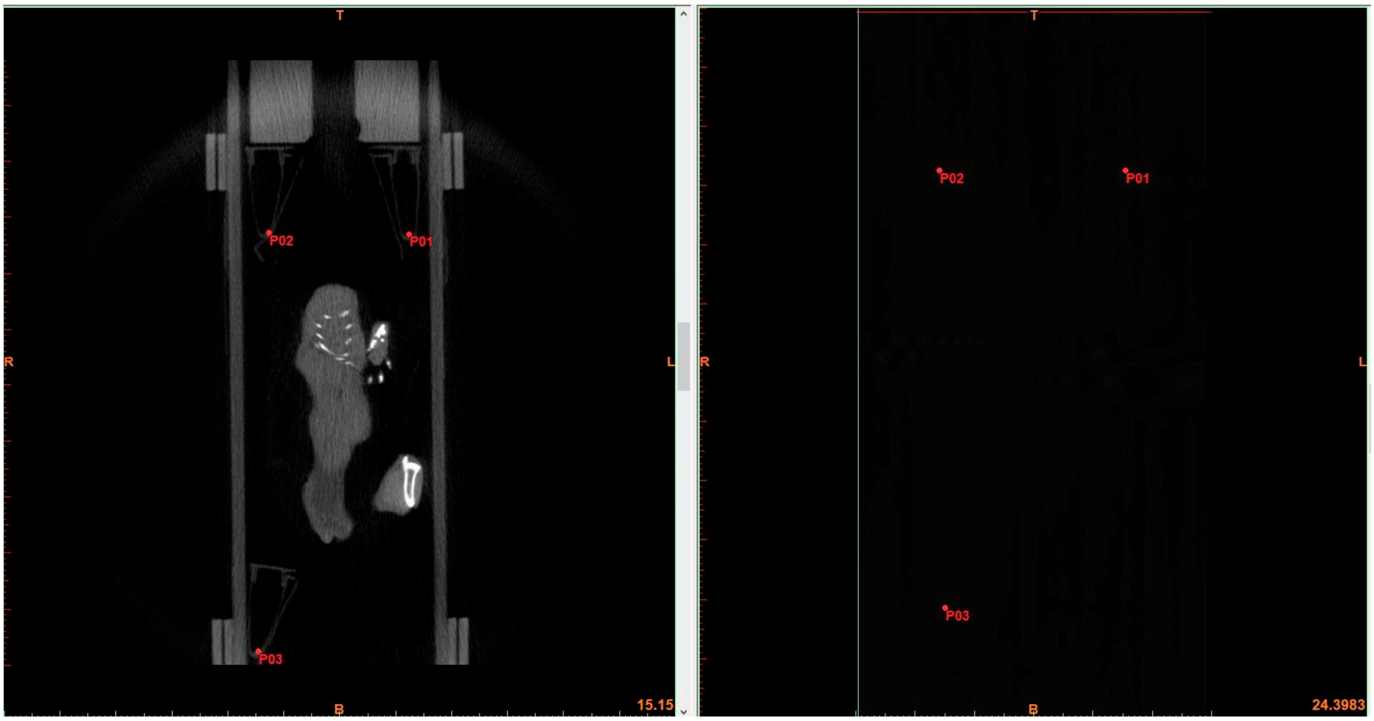

Figure S5. Landmark Registration window with selected fiducial points. .

8. Under the Objects tab, add a new transformation matrix (Figure S6). Manually add the matrix values from the text file created by the Landmark Registration tool.
9. Right click on the MPI image stack in the Project Management tab and apply the newly created transformation matrix.

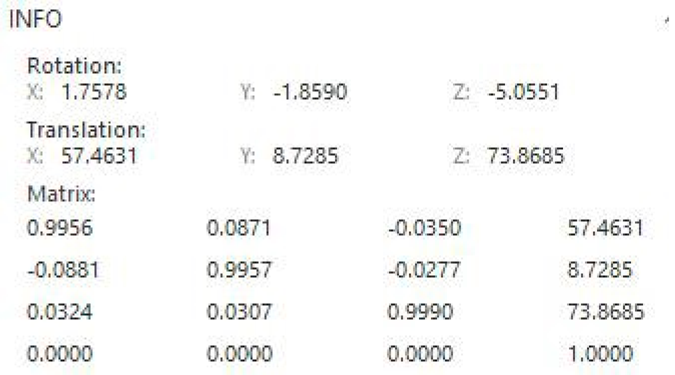
INFO

Rotation:
X: 1.7578 Y: -1.8590 Z: -5.0551

Translation:
X: 57.4631 Y: 8.7285 Z: 73.8685

Matrix:

0.9956	0.0871	-0.0350	57.4631
-0.0881	0.9957	-0.0277	8.7285
0.0324	0.0307	0.9990	73.8685
0.0000	0.0000	0.0000	1.0000

Figure S6. Example transformation matrix in Mimics. .

Creating 3D MNP Distribution from MPI Data

1. Under the Images tab of Project Management, make the MPI file the active image set. Under the Segment menu, select New Mask, and adjust the appropriate threshold bounds based on your application. We recommend creating a large mask to start, since any signal outside the bounds of the tumor will be trimmed regardless.
2. Select the newly created mask under the Objects tab of Project Management. Under the FEA menu, select Create Voxel Mesh. The window (Figure S7) will appear with several options. For COMSOL, select Tetrahedral 4-point mesh. Filtering and Smoothing options are available. We selected default parameters, but the user can adjust these as they see fit. Depending on your computational capabilities and the size of your mask, you can

adjust the resolution in the *X*, *Y* or *Z* dimension to reduce the number of elements. Select Calculate to generate the mesh. The generated mesh can now be overlaid onto the anatomical imaging data.

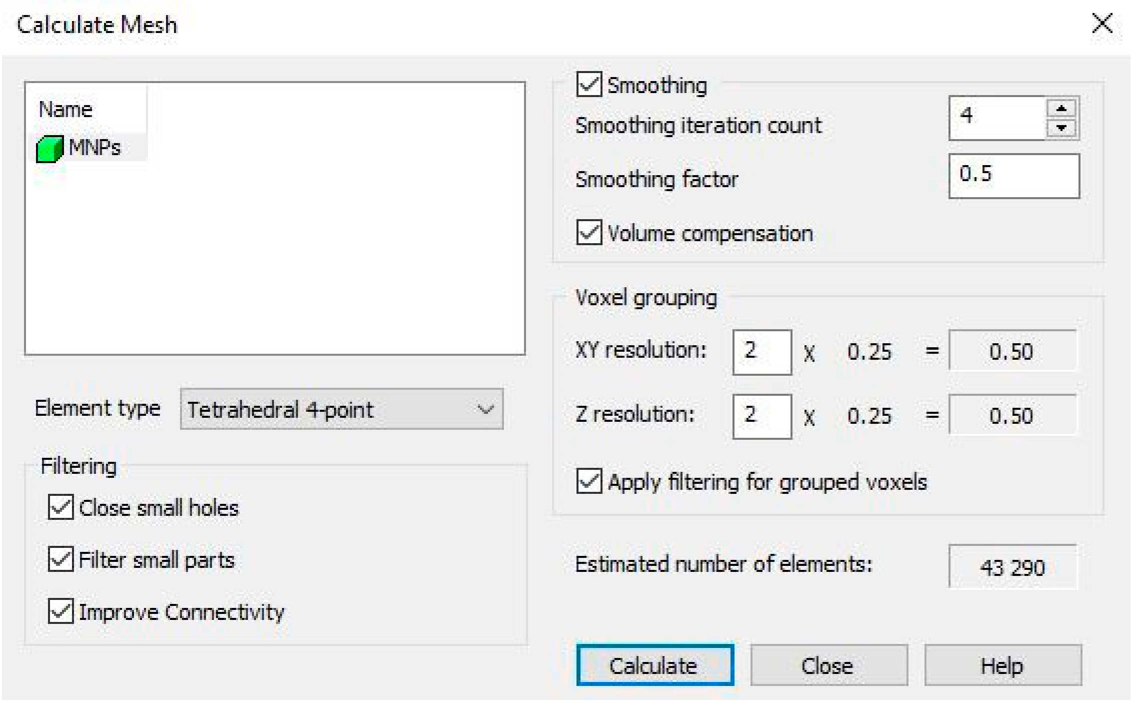

Figure S7. Create Voxel Mesh window.

3. Select the newly created mesh under the Objects tab of Project Management. Under the FEA menu, select Assign Material, where the window (Figure S8) will appear. Select "Gray value based" material assignment method in the top drop-down menu.
4. Use your calibration curve, similar to the one shown in Figure 3 of the main paper, to map the grayscale values within the mesh to their respective thermal output. We recommend using the "density" 2-point menu, where you insert the highest and lowest values from the calibration curve, and Mimics assumes a linear relationship between them. Select apply to implement the calibration curve.
5. Adjust the "Number of Materials" manually until Mimics no longer shows an error in the log after selecting Apply.

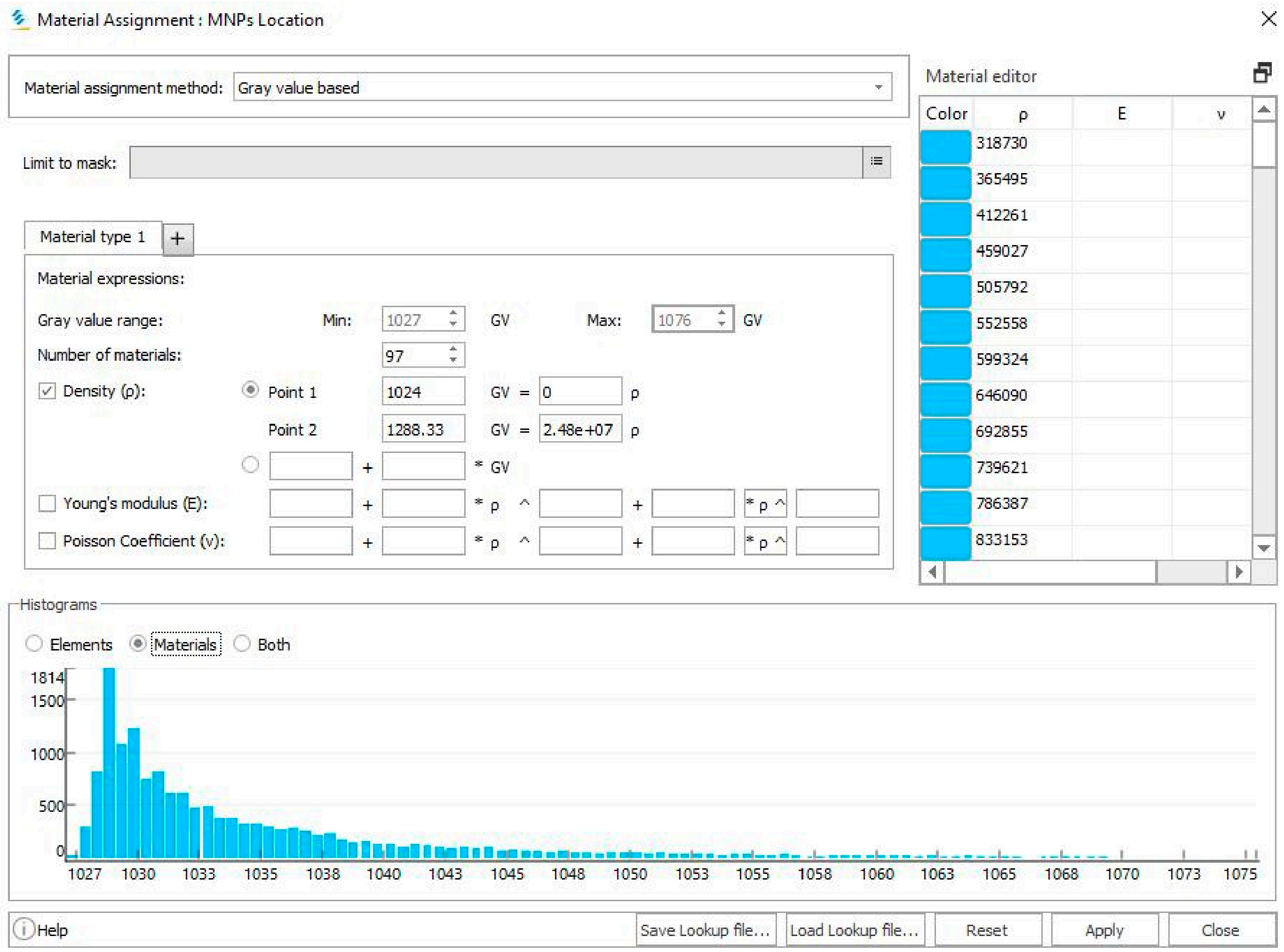

Figure S8. Material Assignment window.

6. Under the File menu, select Export → Meshes → COMSOL. Under the Materials tab, export the MPI mesh as a Material Properties File (.csv).

Creating Tumor Geometry from Anatomical Imagery

1. From the Images tab of Project Management, make the anatomical image stack the active image stack.
2. Under the Segment menu, select New Mask and adjust the window on the image to only include the area around the tumor. Adjust the threshold until the entire tumor is selected.
3. Under the Segment menu, select the Split Mask tool to manually select which regions are tumor vs normal tissue (Figure S9). This will create two new masks.
4. Under the Segment menu, select Calculate Part, select the tumor mask, adjust quality, and select OK.

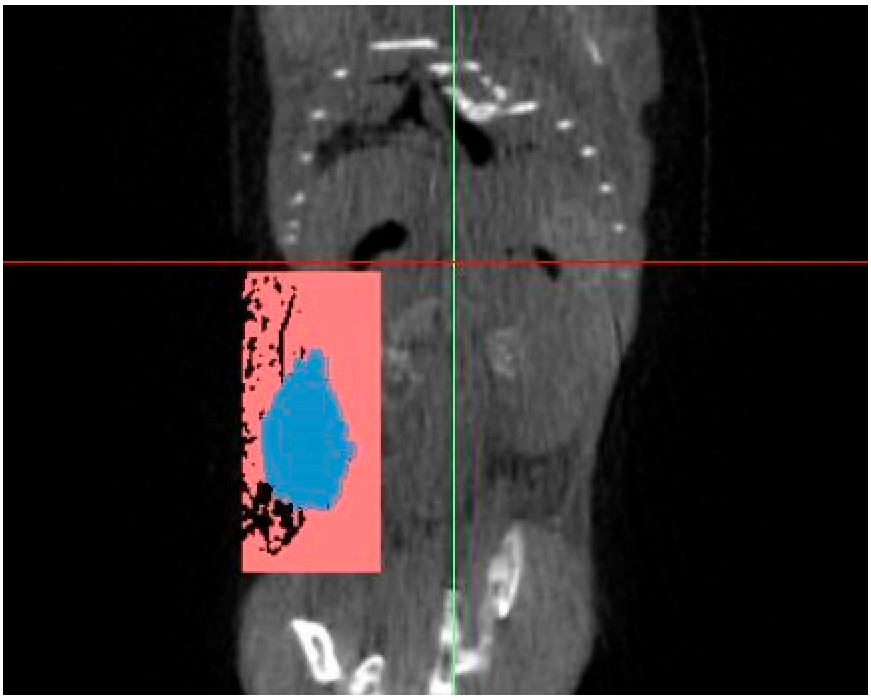

Figure S9. Creating tumor mask.

5. Under the File menu, select Export -> Parts, then pick your file type (we selected .stl). The part will save in your project directory.
6. Open 3-matic. Under the File menu, select Import Part, and select the saved tumor part file.
7. As seen in Figure 4 of the main paper, the tumor segmentation is often very rough and needs to be smoothed to aid physics-based meshing in COMSOL. Under the Fix menu, select Remove Spikes, adjust the parameters, and select Apply. Parameter selection is key and will vary with each tumor. The user will need to optimize parameter selection such that tumor volume is conserved while minimizing the presence of spikes on the part. Spikes create regions of infinitesimally small crosssectional area, which can misrepresent the heat transfer occurring within that region.
8. Under the Remesh menu, select Adaptive Remesh, and select Apply. Then, select Create Volume Mesh, be sure to select Tet4 for COMSOL, and select Apply. These steps will create a surface and volume mesh to be imported into COMSOL.
9. Under the File menu, select Export -> COMSOL, then select Apply. This exports the part as a COMSOL mesh part.

Import Tumor Geometry and MNP Distribution into COMSOL

1. In the Model Builder window, right click on Global Definitions -> Mesh Parts -> 3D mesh parts. Import the tumor geometry mesh part file.
2. Under the Component menu, right click on Geometry, and select Import. Change the source to “Mesh or 3D printing file” and select your imported mesh part. Then, select Build for the tumor geometry to appear. Your tumor geometry is now imported into COMSOL!
3. Right click on Global Definitions -> Functions -> Interpolation. Select File as the data source.
   Import the Material Properties File containing the calibrated MPI data. Select the Number of Arguments to be “3”, select the function units to be “W/m^3” and the Argument units to be “mm”.
   The MPI data can now be spatially referenced within your simulation!
